# Analysing Emotional Well-Being in Cancer Patients: A Natural Language Processing Approach to Correlating Text with Hospital Anxiety and Depression Scale Scores

**DOI:** 10.3390/curroncol33070400

**Published:** 2026-07-04

**Authors:** Mustafa Serkan Alemdar, Hakan Şat Bozcuk

**Affiliations:** 1 Department of Medical Oncology, Istinye University, Istanbul 34396, Turkey; 2 Department of Medical Oncology, Medical Park Hospital, Antalya 07160, Turkey; 3 Department of Medical Oncology, Lara Anatolia Hospital, Antalya 07230, Turkey; hbozcuk@anatoliahospital.com

**Keywords:** artificial intelligence, BERT, cancer, hospital anxiety and depression scale, natural language processing, oncology, psychological distress, sentiment analysis

## Abstract

**Background:** Psychological distress, particularly anxiety and depression, is highly prevalent among cancer patients, and is associated with impaired quality of life, reduced treatment adherence, and increased mortality risk. Standardized screening instruments, such as the Hospital Anxiety and Depression Scale (HADS), are effective, but face implementation barriers in busy oncology outpatient settings. This cross-sectional study investigated whether BERT-based Natural Language Processing (NLP) analysis of brief patient-generated free texts would correlate with HADS scores in a consecutive cohort of cancer outpatients. **Material and Methods:** A total of 165 consecutive adult cancer outpatients were enrolled at a tertiary oncology center in Turkey. All participants completed the HADS questionnaire and were asked to write freely about their current emotional state in Turkish. Patient-generated texts were analyzed using a pre-trained Turkish BERT model to derive a continuous BERT Sentiment Score (BSS) and a categorical BERT Sentiment Cluster (BSC) via unsupervised hierarchical clustering. Univariate and multivariate linear regression analyses were performed to examine associations between clinical, demographic, and NLP-derived variables and the logarithmically transformed HADS score. **Results:** The mean total HADS score was 10.46 (range, 0–33), consistent with a moderate level of psychological distress. In multivariate analysis, two variables were independently associated with HADS scores: female sex (β = 0.20, t = 2.14, *p* = 0.034), associated with higher HADS scores, and BERT Sentiment Score (BSS) (β = −0.18, t = −2.43, *p* = 0.016), with higher values corresponding to lower HADS scores. Hierarchical clustering identified two distinct thematic groups: ‘Coping and Fighting Spirit’ (74%), and ‘Hope and Negative Feelings’ (26%); however, cluster membership (BSC) was not independently associated with HADS scores (β = −0.02, *p* = 0.789). Clinical variables, including cancer stage, diagnosis type, treatment status, and time since diagnosis, also were not independently associated with HADS scores. **Conclusions:** BERT-based sentiment analysis of brief patient-generated free texts yielded a continuous measure that independently correlated with HADS scores in cancer outpatients, alongside female sex. These findings provide proof-of-concept evidence that NLP-derived sentiment scoring may offer a practical, scalable, and complementary approach to standardized psychological screening in routine oncology care.

## Simple Summary

Many people with cancer experience anxiety or depression, but routine emotional screening can be difficult in busy oncology clinics. In this study, 165 adult cancer outpatients completed the Hospital Anxiety and Depression Scale and also wrote a short Turkish free-text statement about how they felt. The written responses were analyzed with an artificial intelligence language model that produced a sentiment score for each patient, reflecting whether the emotional tone was more negative or more positive. More positive sentiment scores were associated with lower anxiety and depression scores, while female patients had higher distress scores. Clinical factors such as cancer stage, diagnosis type, treatment status, and time since diagnosis were not independently linked with distress. These findings suggest that brief patient-written texts, analyzed automatically, may help clinicians identify emotional distress and could complement standard psychological screening in routine oncology care.

## 1. Introduction

Cancer remains a major public health challenge in the contemporary era. According to GLOBOCAN 2022 estimates, there were approximately 20 million new cancer cases and 9.7 million cancer-related deaths worldwide, with global incidence projected to rise to 35 million cases by 2050 [1]. Beyond its physical consequences, a diagnosis of cancer imposes a profound and often persistent psychological burden. Anxiety and depression are among the most common psychiatric comorbidities in patients with cancer. Large-scale meta-analyses have shown that some form of mood disorder affects approximately 30% to 40% of patients across oncology and palliative care settings [2,3]. In addition, the four-week prevalence of any mental disorder across major tumor entities has been estimated at approximately 32% when assessed using standardized diagnostic interviews, with anxiety and adjustment disorders emerging as the most prevalent conditions in this population [4]. Reported prevalence rates vary considerably according to cancer type, disease stage, sex, and age, and women as well as younger patients consistently demonstrate higher levels of emotional distress [5]. More recent evidence reinforces this picture. A 2025 meta-analysis spanning 31 countries confirmed that a substantial proportion of cancer survivors continue to experience clinically relevant anxiety and depression well beyond the diagnostic and acute-treatment phases [6], and an umbrella review of systematic reviews and meta-analyses likewise identified depression and anxiety as among the most frequent and disabling psychological symptoms across cancer populations, while highlighting the considerable heterogeneity in reported prevalence according to assessment method and clinical context [7]. Importantly, depression and anxiety in cancer patients have been associated with poorer quality of life, reduced treatment adherence, and increased mortality risk [8,9,10,11]. In recognition of this burden, international clinical guidelines, including those of the European Society for Medical Oncology (ESMO) and the National Comprehensive Cancer Network (NCCN), emphasize the systematic assessment and management of distress as an essential component of comprehensive cancer care [12,13].

In light of these findings, routine screening for psychological distress is widely recommended as part of good oncological practice. One of the most widely used instruments for this purpose is the Hospital Anxiety and Depression Scale (HADS), developed by Zigmond and Snaith. HADS is a 14-item self-report questionnaire composed of two subscales assessing anxiety and depression, with each item scored on a four-point Likert scale [14]. Its utility in oncology settings is well established. In particular, HADS scores have been shown to correlate strongly with quality-of-life dimensions, and depression assessed by HADS has been reported to be a stronger predictor of impaired global quality of life than anxiety in patients with cancer [10]. Furthermore, the Turkish-language version of HADS has been formally validated and is widely used in Turkish oncology practice [15]. Taken together, these characteristics make HADS a practical and clinically valuable instrument for screening psychological distress in cancer patients, both internationally and within the local national context.

Despite its established validity and clinical usefulness, the routine implementation of structured instruments, such as HADS, in busy oncology outpatient clinics remains challenging. Although distress screening is strongly recommended, it is not consistently integrated into everyday clinical practice [16]. Barriers to widespread implementation include limited consultation time, patient fatigue related to illness and treatment, and the administrative demands associated with systematic psychological assessment. A wide range of screening instruments has been evaluated in oncology settings, and among them, HADS has demonstrated particular utility because of its brevity and its avoidance of somatic symptom items that may overlap with manifestations of cancer or its treatment [16]. Nevertheless, these practical challenges have encouraged growing interest in digital health technologies and artificial intelligence as potential tools for monitoring emotional well-being in routine oncology care.

Natural Language Processing (NLP), a branch of artificial intelligence concerned with the computational analysis of human language, offers a promising approach for the automated assessment of emotional states from patient-generated text. Within the field of NLP, Bidirectional Encoder Representations from Transformers (BERT), introduced by Devlin and colleagues, has emerged as one of the leading architectures for language understanding and has demonstrated remarkable performance across a broad range of tasks, including sentiment analysis [17]. Through its bidirectional attention mechanism, BERT is capable of capturing nuanced contextual information from free-form text, making it particularly suitable for detecting subtle emotional content in patient narratives. In addition, multilingual variants of BERT extend this capability across languages, thereby increasing its potential utility in non-English-speaking clinical settings [17,18].

The application of NLP to patient-reported and clinician-generated oncology data is a rapidly expanding area of research. Recent studies have demonstrated the feasibility of BERT-based models for identifying depression-related concerns in patient portal messages within cancer care [19]. Multimodal models integrating clinical variables with NLP-derived features have also shown reasonable predictive validity for depression risk in large cancer cohorts [20]. Moreover, the application of NLP to documentation from initial oncology consultations has shown that language-based features may help predict which patients subsequently require referral to psychiatric or counseling services [21]. More recently, the rapid emergence of generative large language models (LLMs) has further accelerated this field and broadened the methodological repertoire beyond encoder-only architectures such as BERT. Contemporary work has demonstrated that LLMs and text-embedding approaches can detect depression and even suicidality directly from patient-generated narratives [22], and that carefully prompted models can identify symptoms of depression and anxiety from routine patient messages with high accuracy [23]. Within oncology specifically, recent applied work and expert commentary have begun to examine how such language models might be deployed to flag depression-related concerns in cancer patients [24], underscoring the timeliness of evaluating language-based emotional signals against validated screening instruments. However, although prior studies have applied BERT and related NLP methods to clinically relevant emotional or psychiatric outcomes, we are not aware of studies specifically examining whether BERT-based sentiment scores derived from brief, spontaneous patient narratives correlate directly with validated psychological screening instruments in a prospective clinical oncology setting.

In the present study, we investigated whether short patient-generated texts reflecting the individual’s current emotional state, analyzed using a BERT-based NLP approach, correlate with HADS scores in a consecutive cohort of cancer outpatients. We also examined the contribution of demographic and clinical variables to HADS scores, with the broader aim of evaluating whether NLP-based sentiment analysis may serve as a scalable complement to standardized psychological screening in routine oncology practice.

## 2. Material and Methods

### 2.1. Study Design and Patient Population

This cross-sectional study was conducted in the outpatient oncology clinics of a private hospital’s cancer center. Consecutive adult cancer patients presenting for routine follow-up or treatment appointments were enrolled over the study period. Inclusion criteria were: confirmed cancer diagnosis, age ≥ 18 years, ability to read and write in Turkish, and provision of written informed consent. Patients with cognitive impairment, severe comorbidity precluding meaningful participation, or inability to complete the study instruments were excluded.

### 2.2. Clinical Data Collection

Demographic and clinical data were collected at enrollment, including age, sex, cancer diagnosis, disease stage, active treatment status, time since diagnosis, educational level, income level, and marital status. Age was dichotomized at 60 years for analytical purposes. Cancer diagnoses were grouped into three categories: breast cancer, lung or colorectal cancer, and other cancers. Stage was classified as localized (Stage 1 to 3) or metastatic (Stage 4).

### 2.3. Psychological Assessment

All participants completed the Hospital Anxiety and Depression Scale (HADS) as a tool already validated across diverse clinical populations [25,26]. The HADS comprises 14 items, with seven items assessing anxiety (HADS-A) and seven assessing depression (HADS-D), each scored 0–3, yielding subscale scores of 0–21 and a total score of 0–42. As previously acknowledged, the Turkish-language version of the HADS used in this study has been formally validated for use in Turkish-speaking patients [15]. In this study, the total HADS score was used as the primary outcome measure. Given the skewed distribution of HADS scores, a logarithmic transformation was applied prior to regression analyses.

### 2.4. Free-Text Data Collection

In addition to the HADS questionnaire, participants were asked to write freely about their current feelings and emotional state. Participants’ writings were collected at outpatient clinics in association with their routine treatments and physician appointments. No minimum or maximum length was specified; participants were encouraged to express themselves in as many or as few sentences as they wished. Written texts were collected in Turkish and subsequently processed using NLP methods.

### 2.5. Natural Language Processing Analysis

BERT-based sentiment analysis was performed on the patient-generated free texts using the Transformers library. A BERT model pretrained for sentiment classification was applied to each sentence in the participant’s text. The BERT Sentiment Score (BSS) for each participant was computed as a continuous composite score reflecting the overall sentiment valence of their free text, ranging from −1.00 (most negative) to +1.00 (most positive). The cleaned text data were passed through a pre-trained BERT model specific to Turkish (dbmdz/bert-base-turkish-cased; https://huggingface.co/dbmdz/bert-base-turkish-cased (accessed on 1 January 2025)) to obtain the embeddings.

Because patient-generated narratives varied in length and level of expressive detail, the analysis was performed at the sentence level before deriving a participant-level score. Each text was segmented into sentences, and the BERT-based sentiment model was applied to each sentence separately; these sentence-level outputs were then combined into a single BERT Sentiment Score (BSS) for each participant. In this way, both short and longer narratives contributed one standardized sentiment measure per patient, reducing the likelihood that text length alone would drive the analysis. Differences in narrative depth and writing quality were treated as part of the natural variability of spontaneous outpatient free-text responses, while the final analyses were based on participant-level BSS and BERT Sentiment Cluster rather than raw text volume.

For clustering analysis, unsupervised hierarchical clustering was applied to the BERT-derived feature representations. The optimal number of clusters was determined by visual inspection of the dendrogram and internal cluster validity criteria.

### 2.6. Statistical Analysis

Descriptive statistics were reported as frequencies and percentages for categorical variables and as means, medians, and ranges for continuous variables. Univariate linear regression analyses were conducted to examine the associations between individual clinical and textual predictors and the logarithmically transformed HADS score. Variables with *p* < 0.10 in univariate analysis, along with clinically relevant covariates, were entered into a multivariate linear regression model using a backward elimination approach. Standardized beta coefficients (β), *t*-statistics, and *p* values were reported. Statistical significance was defined as *p* < 0.05. All analyses were performed using Python (version 3) with the scikit-learn, scipy, and statsmodels libraries, within the Google Colab environment. Additionally, SPSS 21 software was used for the regression analysis.

The study was conducted in accordance with the Declaration of Helsinki. Ethical approval was obtained from the Medical Park Antalya Hospital Ethics Committee (Approval No: 2023/17, Date: 17 December 2023) prior to study initiation. All participants provided written informed consent prior to enrollment. Participants did not receive individual AI-generated feedback or BERT-based reports, and the BERT outputs were not used for clinical decision-making. The NLP analysis was performed for research purposes only. Participants were informed that their written responses would be scientifically evaluated in relation to emotional well-being.

## 3. Results

### 3.1. Patient Characteristics

A total of 165 consecutive cancer outpatients were enrolled in the study. The median age was 61 years (range, 31–81), and the cohort was evenly split between those aged ≤60 years (*n* = 82, 50%) and those aged >60 years (*n* = 83, 50%). The majority of participants were female (*n* = 101, 61%). The demographic and clinical characteristics of the cohort and summary data for the textual analysis and HADS scores are presented in Table 1.

### 3.2. HADS Scores, Textual Data and NLP Analysis

The mean total HADS score was 10.46 (median = 10, range = 0–33), indicating a moderate level of psychological distress in this cohort. The distribution of HADS scores was positively skewed, necessitating logarithmic transformation for regression analysis.

**Figure 1:**
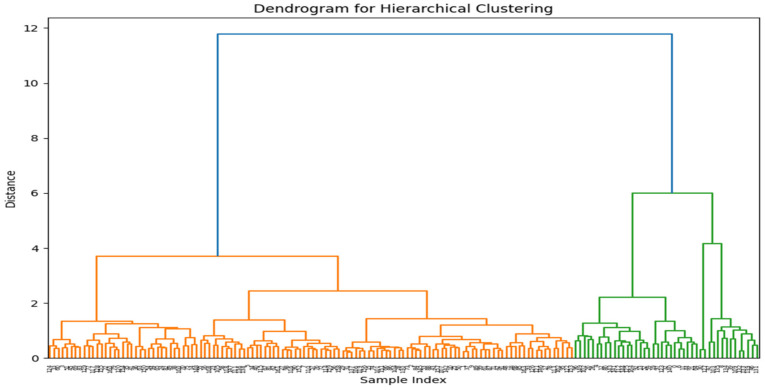
Dendrogram showing hierarchical clustering of BERT-derived textual features.

**Figure 2:**
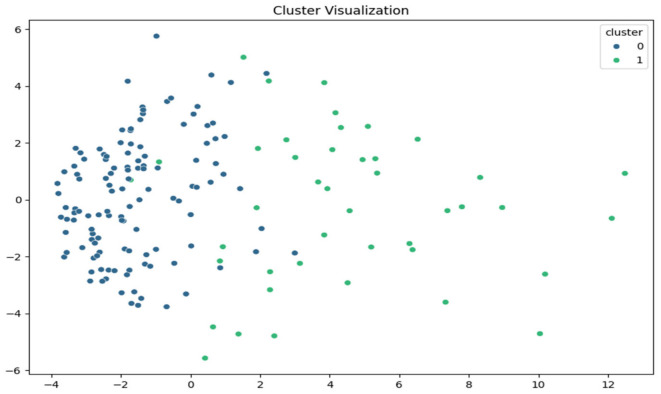
Distribution of BERT sentiment clusters among patients.

Participants generated a mean of 2.34 sentences (median = 2, range = 0–10). Hierarchical clustering of the BERT-derived textual features identified two distinct clusters (Figure 1 and Figure 2). The larger cluster (Cluster 0, *N* = 122, 74%) was characterized by themes of active coping, resilience, and fighting spirit, and was accordingly labeled “Coping and Fighting Spirit.” The smaller cluster (Cluster 1, *N* = 43, 26%) was characterized by themes of hope intermingled with negative emotions and apprehension, and was labeled “Hope and Negative Feelings.”

### 3.3. Regression Analyses

In the univariate analysis, age (β = −0.17, *p* = 0.034), sex (β = 0.26, *p* = 0.001), and BERT Sentiment Score (β = −0.21, *p* = 0.008) were significantly associated with HADS score. Subsequently, in the multivariate analysis, two variables remained independently associated with HADS scores: sex (male vs. female; β = 0.20, t = 2.14, *p* = 0.034) and BERT Sentiment Score (β = −0.18, t = −2.43, *p* = 0.016). Female sex was associated with higher HADS scores, and higher (more positive) BERT Sentiment Scores were associated with lower (better) HADS scores, independent of other covariates. In addition, BERT Sentiment Cluster was not independently associated with the HADS score (β = −0.02, *p* = 0.789). Table 2 presents the results of univariate and multivariate regression analyses examining correlates of the logarithmically transformed HADS score.

**Figure 3:**
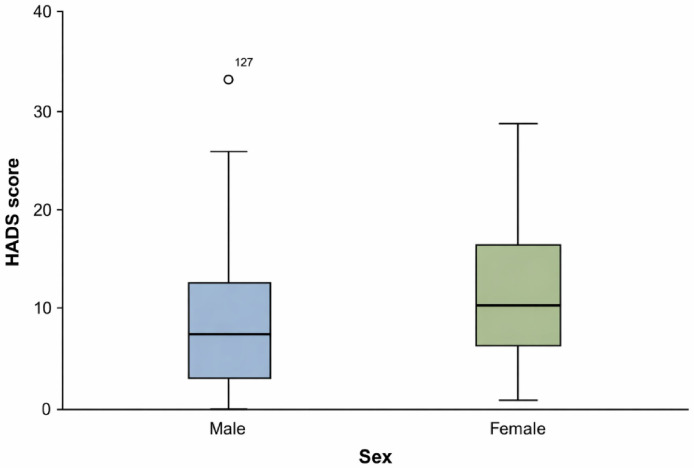
Association between sex and HADS score.

**Figure 4:**
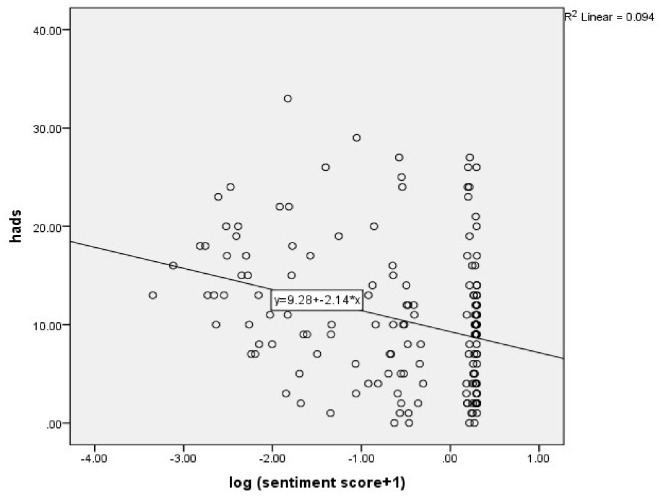
Association between BERT Sentiment Score and HADS score. “*” signifies multiplication.

The associations of the significant factors from the multivariate regression analyses (female gender and logarithmic transformation of BSS) with HADS scores are shown in Figure 3 and Figure 4.

## 4. Discussion

To the best of our knowledge, this is among the first cross-sectional studies to systematically correlate BERT-based natural language processing (NLP) analysis of brief, patient-generated free texts with a validated psychological screening instrument in an oncology outpatient setting. The principal finding of this study is that the BERT Sentiment Score (BSS)—a continuous, NLP-derived measure of emotional valence extracted from patients’ written narratives—exhibited independent association with HADS scores, with this association being controlled for relevant clinical and demographic variables. Secondly, multivariate analysis revealed a significant independent association between female sex and elevated HADS scores, as already demonstrated previously [5]. In contrast, clinical variables, including cancer stage, diagnosis category, active treatment status, and time since diagnosis, were not independently associated with HADS scores. The BERT Sentiment Cluster (BSC) variable also did not reach statistical significance.

The mean total HADS score in our cohort was 10.46, indicating a moderate level of psychological distress. This figure is consistent with the findings of previous studies that reported moderate levels of psychological distress in a non-selected population of cancer outpatients [2,12]. A significant proportion of cancer patients—approximately 30 to 40 percent—exhibit clinically significant mood disorders, underscoring the necessity for systematic screening in this population [2]. A notable issue is the undertreatment of depression in cancer patients within standard clinical practice. However, there is sufficient evidence to support the efficacy of evidence-based psychosocial interventions when patients are accurately identified and referred [27,28]. The distribution of HADS scores in our sample exhibited positive skew and necessitated logarithmic transformation prior to regression analyses. This distribution reflects the heterogeneity of psychological distress within oncology populations, and is a recognized feature of HADS data in clinical research [25,26].

The central and novel finding of this study is the independent association between BSS and HADS scores in multivariate analyses. A higher BSS, indicative of more positive or optimistic sentiment in patients’ written narratives, was associated with lower HADS scores, suggesting reduced psychological distress. This directionally consistent relationship lends support to the hypothesis that BERT-based sentiment analysis of brief, free-form patient texts captures genuine psychometric signals that correlate with standardized measures of anxiety and depression. Studies employing natural language processing (NLP) on patient portal messages in oncology settings have yielded comparable findings. In these studies, BERT-based classifiers have achieved Area Under the Curve (AUROC) values ranging from 0.86 to 0.88 in identifying depression-related concerns [19]. The present study extends this line of evidence to a direct, regression-based correlation with a validated, continuous distress measure, using spontaneous patient narratives rather than asynchronous electronic messages. Consistent with our observations, transformer-based sentiment approaches have continued to demonstrate value across diverse healthcare contexts; a recent comparison of BERT with lexicon-based methods on patient-authored narratives found that transformer architectures aligned more closely with patient-reported experience than traditional dictionary approaches [29]. At the same time, the broader migration from encoder-only models such as BERT toward generative large language models has expanded the toolkit available for narrative-based emotional assessment, with recent studies reporting strong performance in detecting depression, anxiety, and related distress directly from free-text patient communications [22,23]. These parallel developments situate the present findings within a rapidly maturing body of evidence and suggest that the continuous sentiment scoring approach examined here is well positioned to benefit from ongoing methodological advances.

The BSC variable, a binary categorical grouping derived from hierarchical clustering of BERT-derived textual features, did not demonstrate a significant association with HADS scores (*p* = 0.789). This null finding is informative. The two identified clusters, designated ‘Coping and Fighting Spirit’ and ‘Hope and Negative Feelings,’ based on a qualitative examination of cluster content, represent qualitatively distinct thematic orientations in patients’ emotional expression. However, both clusters appear to encompass a wide range of HADS scores, suggesting that qualitative emotional themes and continuous distress severity are not straightforwardly aligned. The continuous BSS measure appears to capture more granular variance in emotional tone than the binary cluster assignment, which may explain its superior predictive utility. These observations are consistent with findings from multimodal NLP studies in oncology, which have similarly found that continuous NLP-derived features outperform categorical text classifications in predicting clinical outcomes [20].

Gender disparities in psychological distress among cancer patients are among the most consistently replicated findings in the field of psycho-oncology. Female patients report higher rates of anxiety and depression across cancer types, treatment stages, and cultural contexts [5,10]. Anxiety disorders specifically impair the quality of life of cancer patients, regardless of disease stage [30]. In the present cohort, female sex was independently associated with higher HADS scores (β = 0.20, *p* = 0.034) in multivariate analysis, in accordance with the previous literature [5]. The proposed mechanisms encompass a variety of factors, including variations in emotional coping styles, increased propensity to disclose psychological symptoms, hormonal influences, and the predominance of breast cancer—a diagnosis that is associated with comparatively higher psychological distress—in female cohorts [31]. The replication of this well-established gender association in our dataset provides additional confidence in the validity of our study cohort and analytical approach.

The absence of significant associations between clinical variables—including cancer stage, diagnosis type, active treatment status, and time since diagnosis—and HADS scores in multivariate analyses may appear counterintuitive. Nevertheless, this finding is in accordance with previous research that has shown that the degree of psychological distress in cancer patients is not directly predicted by objective clinical characteristics alone [2,12]. A considerable amount of variance in outcomes related to psychological distress is attributable to individual psychological resources, resilience, social support, and prior psychiatric history [32,33,34]. The ESMO guideline similarly acknowledges the non-linear relationship between disease severity and psychological morbidity, which is moderated by a range of psychosocial factors [12]. These observations underscore the significance of employing individualized, psychological screening methods that do not depend exclusively on clinical staging to identify patients at risk.

The NLP-based methodology employed in this study offers several potential, practical advantages over conventional, structured psychological screening. Firstly, the free-text task is brief, naturalistic, and less onerous for patients than completing multi-item questionnaires during busy outpatient visits. Secondly, the implementation of automated, BERT-based sentiment analysis can be executed expeditiously and on a large scale without necessitating the allocation of additional clinical staff time beyond that allocated for data collection. Thirdly, Turkish-specific and multilingual BERT architectures have the potential to be applied across different linguistic contexts, thereby broadening their applicability to diverse clinical populations. The incorporation of artificial intelligence (AI)-driven natural language processing (NLP) tools into oncology workflows signifies a burgeoning domain within the realm of clinical informatics research. NLP-based models have already exhibited clinical utility in predicting psychiatric referral needs and depression risk in substantial cancer center datasets [20,21]. Additionally, BERT-like procedures could theoretically be of value in assessing clinical notes and reports generated by nurses and physicians, allowing a deeper analysis of patients’ symptoms. The present study contributes cross-sectional validation evidence from a distinct healthcare context employing a different textual data source. Specifically, it utilizes brief elicited outpatient narratives, as opposed to clinical notes or online messages. A recent scoping review of empathic AI in oncology reached convergent conclusions, noting that systems combining clinical precision with emotional intelligence hold considerable promise for distress recognition and psychosocial support, while cautioning that responsible integration must address the risks of overreliance on automated tools, cultural insensitivity, and patient privacy [35]. These considerations are directly relevant to the prospective deployment of NLP-based sentiment measures such as the one evaluated here, which are intended to complement rather than replace clinician judgment and validated screening.

Several limitations of the present study should be acknowledged. Firstly, this was a single-center study conducted at a private hospital’s oncology outpatient clinic. The generalizability of our findings to other clinical settings, patient populations, and healthcare systems requires further evaluation. Secondly, the cross-sectional study design precludes assessment of longitudinal changes in BSS or their temporal relationship with HADS score trajectories over the course of illness and treatment. Thirdly, another potential limitation is that educational level and individual communication ability may have influenced the quality and linguistic richness of the free-text responses. Patients with higher literacy or stronger expressive skills may have produced more nuanced narratives, allowing the BERT-based model to capture emotional valence more accurately, whereas shorter, less structured, or less expressive texts may have provided a weaker signal. Therefore, part of the variability in NLP-derived sentiment scores may reflect not only emotional state but also differences in patients’ ability to verbalize their feelings in written form. Notwithstanding these limitations, our findings provide proof-of-concept evidence for the clinical utility of BERT-based natural language processing (NLP) analysis of brief patient-generated free texts as a correlate of standardized psychological distress measurement in oncology. Future research should examine the longitudinal validity of NLP-derived sentiment measures, their sensitivity to treatment-related changes in psychological status, and their incremental value over standard clinical predictors in larger, prospective, multicenter cohorts.

## 5. Conclusions

In this cross-sectional study of 165 consecutive cancer outpatients, the BSS, derived from brief patient-generated free texts, and female sex were independently associated with total HADS scores after multivariate adjustment. The BERT Sentiment Cluster exhibited qualitatively distinct thematic content; however, it did not independently predict HADS scores. These findings suggest that NLP-based analysis of short patient narratives may offer a practical, scalable, and complementary approach to monitoring emotional well-being in oncology settings, with potential implications for the integration of artificial intelligence tools into routine cancer care.

## Figures and Tables

**Table 1:** Clinical and textual characteristics of the study cohort (*n* = 165).

Variable	Category	*n* (%)	Range	Median	Mean
**Clinical characteristics**					
Age			31–81	61	59
	Up to 60 years	82 (50)			
	More than 60 years	83 (50)			
Sex	Male	64 (39)			
	Female	101 (61)			
Diagnosis	Breast cancer	63 (38)			
	Lung or colorectal cancer	46 (28)			
	Other cancers	56 (34)			
Stage	Stage 1 to 3	87 (53)			
	Stage 4	78 (47)			
Active treatment	Yes	56 (34)			
	No	109 (66)			
Time since diagnosis	Less than 6 months	37 (22)			
	6 to 12 months	27 (17)			
	More than 12 months	101 (61)			
Education	Primary or secondary school	59 (36)			
	Lycee	35 (21)			
	University	71 (43)			
Income	Poor	25 (15)			
	Medium	128 (78)			
	High	12 (7)			
Marital status	Single	35 (21)			
	Married	130 (79)			
HADS score			0–33	10	10.46
**Textual characteristics**					
Total sentence count			0–10	2	2.34
BERT sentiment score *			−1.00 to 1.00	0.52	0.02
BERT sentiment cluster **	Coping and fighting spirit	122 (74)			
	Hope and negative feelings	43 (26)			

*; Transformer Bert model score, **; Transformer Bert model hierarchical cluster.

**Table 2:** Univariate and multivariate linear regression analyses of variables associated with HADS scores.

Variable	Comparison	Univariate Analyses			Multivariate Analyses		
		β *	t	*p*	β *	t	*p*
Clinical variables							
Age	Up to 60 years vs. older	−0.17	−2.14	0.034	−0.04	−0.48	0.633
Sex	Male vs. female	0.26	3.40	0.001	0.20	2.14	0.034
Diagnosis-1 **	Breast cancer vs. other cancers	−0.05	−0.56	0.574	—	—	—
Diagnosis-2 **	Lung or colorectal cancer vs. other cancers	−0.15	−1.75	0.082	−0.05	−0.61	0.544
Stage	Stages 1–3 vs. 4	−0.12	−1.58	0.117	−0.04	−0.46	0.645
Active treatment	Yes vs. no	−0.06	−0.82	0.414	—	—	—
Time since diagnosis	<6 vs. 6–12 vs. >12 months	−0.02	−0.26	0.795	—	—	—
Education	Primary/secondary vs. lycee vs. university	−0.06	−0.79	0.429	—	—	—
Income	Poor vs. medium vs. high	−0.08	−0.99	0.323	—	—	—
Marital status	Single vs. married	0.03	0.43	0.668	—	—	—
Textual variables							
BERT sentiment cluster £	Coping and fighting spirit vs. hope and negative feelings	−0.02	−0.27	0.789	—	—	—
BERT sentiment score #	Continuous score	−0.21	−2.71	0.008	−0.18	−2.43	0.016

*; Standardized beta coefficient, **; Dummy variables entered together, £; Transformers Bert model hierarchical cluster, #; Transformers Bert model score.

## Data Availability

The raw data supporting the conclusions of this article will be made available by the authors on request.
